# Impact of Intraoperative Nefopam on Postoperative Pain, Opioid Use, and Recovery Quality with Parietal Pain Block in Single-Port Robotic Cholecystectomy: A Prospective Randomized Controlled Trial

**DOI:** 10.3390/medicina60060848

**Published:** 2024-05-23

**Authors:** So Yeon Lee, Dong Hyun Kim, Jung Hyun Park, Min Suk Chae

**Affiliations:** 1 Department of Anesthesiology and Pain Medicine, Eunpyung St. Mary’s Hospital, College of Medicine, The Catholic University of Korea, Seoul 03312, Republic of Korea; soyeon0719@catholic.ac.kr (S.Y.L.); moocimay@naver.com (D.H.K.); 2 Department of Surgery, Eunpyung St. Mary’s Hospital, College of Medicine, The Catholic University of Korea, Seoul 03312, Republic of Korea; angle49@catholic.ac.kr; 3 Department of Anesthesiology and Pain Medicine, Seoul St. Mary’s Hospital, College of Medicine, The Catholic University of Korea, Seoul 06591, Republic of Korea

**Keywords:** nefopam, rectus sheath block, postoperative analgesia, single-port robot-assisted laparoscopic cholecystectomy

## Abstract

*Background and Objectives*: This study explored how nefopam, a non-opioid analgesic in a multimodal regimen, impacts postoperative pain, opioid use, and recovery quality in single-port robot-assisted laparoscopic cholecystectomy (RALC) patients with a parietal pain block, addressing challenges in postoperative pain management. *Materials and Methods*: Forty patients scheduled for elective single-port RALC were enrolled and randomized to receive either nefopam or normal saline intravenously. Parietal pain relief was provided through a rectus sheath block (RSB). Postoperative pain was assessed using a numeric rating scale (NRS) in the right upper quadrant (RUQ) of the abdomen, at the umbilicus, and at the shoulder. Opioid consumption and recovery quality, measured using the QoR-15K questionnaire, were also recorded. *Results*: The 40 patients had a mean age of 48.3 years and an average body mass index (BMI) of 26.2 kg/m^2^. There were no significant differences in the pre- or intraoperative variables between groups. Patients receiving nefopam reported significantly lower RUQ pain scores compared to the controls, while the umbilicus and shoulder pain scores were similar. Rescue fentanyl requirements were lower in the nefopam group in both the PACU and ward. The QoR-15K questionnaire scores for nausea and vomiting were better in the nefopam group, but the overall recovery quality scores were comparable between the groups. *Conclusions*: Nefopam reduces RUQ pain and opioid use post-single-port RALC with a parietal pain block without markedly boosting RSB’s effect on umbilicus or shoulder pain. It may also better manage postoperative nausea and vomiting, underscoring its role in analgesia strategies for this surgery.

## 1. Introduction

Acute cholecystitis, characterized by right upper quadrant (RUQ) abdominal pain, nausea, vomiting, and fever, often requires surgical intervention. Laparoscopic cholecystectomy is the preferred method due to its low conversion rates and minimal bile duct injury risk [1,2]. Robotic surgery, with advanced features like wristed instruments, improved ergonomics, tremor reduction, and enhanced visualization, addresses many limitations of traditional laparoscopy and offers significant advantages in complex procedures, such as bile duct exploration and repair [3,4]. Despite these benefits, patients undergoing robot-assisted laparoscopic cholecystectomy (RALC) still experience significant postoperative pain. Effective pain management in these minimally invasive surgeries is essential for enhancing recovery and reducing opioid dependence, which is associated with adverse effects and risk of dependency [5,6].

Postoperative laparoscopic cholecystectomy pain can arise from surgical incisions, pneumoperitoneum, and visceral sources, leading to extended recovery, delayed discharge, and higher readmission rates [7,8,9,10]. Shim et al. reported that a multimodal approach combining parietal pain blocks with non-opioid analgesics significantly improved recovery, reducing peak pain, the need for additional analgesics, and postoperative nausea and vomiting (PONV) [11]. Single-port RALC offers advantages over traditional methods by minimizing pain and complications through a single small incision [12]. However, certain unavoidable aspects of surgery can still cause pain. Current multimodal pain management, including the Enhanced Recovery after Surgery (ERAS) protocol with nerve blocks and non-opioid analgesics, is effective, but the specific benefits of parietal pain blocks and visceral pain relief remain to be fully explored [13]. 

Nefopam is a unique non-opioid painkiller, originally derived from the non-sedative benzoxazocine, that targets the central nervous system, specifically the brain and spinal cord, to relieve pain through various mechanisms. It primarily works by increasing the levels of serotonin, norepinephrine, and dopamine and decreasing glutamate release by adjusting the activity of calcium and sodium channels [14]. A study of rats demonstrated that nefopam affects N-methyl-D-aspartic acid (NMDA) receptors, reduces c-Fos expression in the spinal cord dorsal horn, and alleviates allodynia, a condition in which ordinary stimuli cause pain [15]. Nefopam reduces the need for morphine by 30–50%, indicating a significant morphine-sparing effect [16,17]. Comparisons between nefopam and opioids have shown that 20 mg nefopam provides similar postoperative pain relief to 12 mg morphine [18]. Furthermore, nefopam is not only effective for managing postoperative pain but also has significant effects on neuropathic pain, which can be challenging to address with conventional medications [19,20,21,22]. The success of nefopam at treating neuropathic pain is attributed to its multifaceted mechanism of action. This includes acting as an antidepressant and anticonvulsant, blocking NMDA glutamate receptors, reducing sympathetic nervous system activity, inhibiting calcium influx and the formation of intracellular cyclic guanosine monophosphate, and preventing the activation of voltage-sensitive calcium channels that can lead to NMDA receptor-dependent neurotoxicity [23,24]. These diverse mechanisms contribute to the efficacy of nefopam in pain management, offering an alternative to opioids with a different, potentially beneficial profile for patients undergoing surgery.

Incorporating nefopam into multimodal analgesia for major surgery reduces postoperative pain and morphine consumption [14]. In patients undergoing orthopedic surgery such as hip arthroplasty, nefopam showed significant morphine-sparing effects, as well as lower side effects and postoperative pain scores, particularly for patients with intense preoperative pain [22]. Furthermore, a recent study of patients who had a percutaneous endoscopic lumbar discectomy found that nefopam significantly alleviated neuropathic pain symptoms such as paresthesia and dysesthesia [19]. Despite these findings, the analgesic impact of nefopam on visceral, parietal, and referred pains specifically in the context of single-port RALC, particularly when combined with a parietal pain rectus sheath block (RSB), remains underexplored.

Therefore, this prospective randomized controlled trial assessed the effects of nefopam on various pain scores and opioid use in the post-anesthesia care unit (PACU) and its influence on the quality of recovery as reported by patients after undergoing single-port RALC.

## 2. Patients and Methods

### 2.1. Ethical Considerations

This prospective randomized trial was approved by the Institutional Review Board and Ethics Committee of Eunpyeong St. Mary’s Hospital, Catholic University of Korea, a tertiary academic teaching hospital, on 17 November 2022 (approval number: PC22MASI0189). The study protocol was registered in advance with the Clinical Research Information Service, a site endorsed by the International Committee of Medical Journal Editors, in the Republic of Korea (approval number: KCT0008281). Adhering to the principles of the Declaration of Helsinki, informed consent was secured from all participants the day before surgery, which took place from 1 March to 6 October 2023. The research followed the Consolidated Standards of Reporting Trials guidelines, ensuring adherence to high ethical and methodological standards.

### 2.2. Study Population

The trial targeted patients aged 19 to 75 years, with an American Society of Anesthesiologists (ASA) physical status of I or II, who were slated for elective single-port RALC. Exclusion criteria included refusal to participate; an ASA classification of III or higher; a history of previous abdominal surgery; emergency surgery, reoperations, or conversion to traditional laparoscopic/open surgery. Patients experiencing intraoperative hemodynamic instability due to significant bleeding or those requiring blood transfusion were also excluded.

Of 44 eligible subjects, 4 were excluded: 3 because of prior abdominal surgery and 1 due to refusal to participate. As a result, 40 patients were enrolled and equally distributed into two groups; 20 patients received nefopam and 20 did not.

### 2.3. Randomization and Blinding

**Figure 1:**
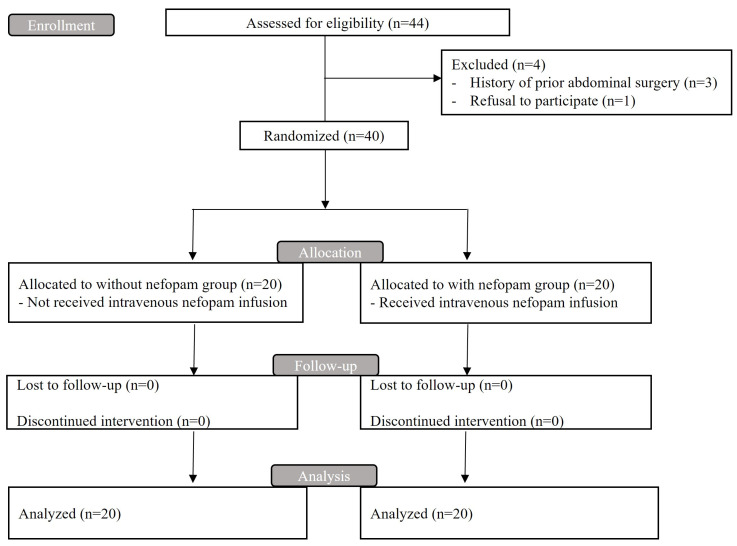
Flow diagram of this study.

The patients were assigned to either the normal saline or the nefopam group through stratified block randomization using a web-based generator (www.random.org, accessed on 2 January 2023). This involved research staff opening sequentially numbered opaque envelopes that designated each patient’s group. This allocation occurred in the surgery waiting room and was communicated to the drug preparation room via a sealed envelope. An anesthesia nurse, who did not participate in evaluating the outcomes, prepared the medications according to the assigned numbers. The normal saline and nefopam solutions were made to look identical to ensure blinding and then were delivered to the operating room. To preserve impartiality, all anesthesiologists and healthcare providers responsible for assessing the postoperative outcomes remained blind to the group assignments (Figure 1).

### 2.4. Study Protocol

#### 2.4.1. Surgery and General Anesthesia

Single-port RALC with the da Vinci Xi system, as per the guidelines of Intuitive Surgical (Sunnyvale, CA, USA), starts with the patient in the lithotomy position under general anesthesia. A 2.0–2.5 cm midline intra-umbilical incision is made down to the fascia using standard surgical techniques. A multichannel port is then inserted, and pneumoperitoneum is established, with the intra-abdominal pressure set at 8 mmHg to facilitate the docking procedure. Proper trocar alignment follows, and a laparoscopic grasper is introduced through an accessory cannula so the first assistant, positioned at the patient’s crotch, can retract the gallbladder fundus upward and toward the right shoulder. The cholecystectomy proceeds retrogradely, using a Cadiere grasper, dissector, and hook, with the cystic duct and artery secured by Hem-o-lok^®^ clips (Teleflex Medical, Morrisville, NC, USA). Once the gallbladder is detached from the liver bed, the da Vinci Xi is undocked, and all instruments and the cannula are removed under vision. The gallbladder is extracted through the incision using a laparoscopic grasper inserted through the accessory port. The fascial defect is closed with 0 Vicryl^®^ (Ethicon, Cincinnati, OH, USA), and the skin is sutured subcuticularly with 4–0 Monosin^®^ (B.Braun, Melsungen, Germany).

Balanced anesthesia was administered by experienced anesthesiologists. Anesthesia induction utilized 1–2 mg/kg propofol (Fresenius Kabi, Bad Homburg, Germany) and 0.6 mg/kg rocuronium (Merck Sharp & Dohme Corp., Kenilworth, NJ, USA). Anesthesia was maintained with 2.0–6.0% desflurane (Baxter, Deerfield, IL, USA) in a mixture of medical air and oxygen. Continuous infusion of remifentanil (Hanlim Pharm. Co., Ltd.; Seoul, Republic of Korea) was performed at a rate of 0.1–0.5 μg/kg/min from the time of inducing anesthesia prior to tracheal intubation throughout the surgery, adjusted by the anesthesiologists. The depth of the anesthesia was monitored using the Bispectral IndexTM (Medtronic, Minneapolis, MN, USA), targeting a range of 40 to 50. Rocuronium was administered as needed, guided by train-of-four monitoring, ensuring more than one twitch. Ventilator settings were adjusted to maintain end-tidal CO_2_ levels between 30 and 40 mmHg. At the end of the procedure, 4 mg/kg sugammadex (Merck Sharp & Dohme Corp., Kenilworth, NJ, USA) was administered to reverse the neuromuscular blockade, followed by 100% oxygen.

Postoperative analgesia comprised 50 µg fentanyl IV for peak numeric rating scale (NRS) scores >6 or upon patient request, as determined by anesthesiologists in the PACU and physicians in the ward, none of whom were involved in the trial. No preemptive pain medication was used during the perioperative period to directly evaluate the nefopam’s analgesic efficacy. Only 50 µg of fentanyl was selected as the rescue analgesic to evaluate its impact on opioid consumption. Nursing staff recorded all analgesic medications.

#### 2.4.2. Nefopam Administration

Nefopam was prepared by mixing 40 mg (20 mg/2 mL nefopam hydrochloride from two vials; Myungmoon Pharm, Seoul, Republic of Korea) with 100 mL 0.9% normal saline [25]. In parallel, the control solution consisted of 100 mL 0.9% normal saline alone. Each patient was administered a single dose of their assigned solution through a slow intravenous infusion over 30 min, concluding immediately before skin closure.

#### 2.4.3. Performance of RSB for Parietal Pain Relief

Immediately after the initiation of general anesthesia, a RSB was performed by an experienced attending anesthesiologist who was not part of this study. Using ultrasound guidance, the probe was placed horizontally above the umbilicus on the rectus abdominis muscle; then, a 22 G Tuohy-type epidural needle was precisely maneuvered in-plane from a mediolateral direction to the point where the muscle meets the posterior sheath, taking care to avoid any nearby blood vessels. Upon confirmation that no blood was aspirated, 20 mL 0.375% ropivacaine was injected. Then, this process was mirrored on the other side of the abdomen.

#### 2.4.4. Postoperative Pain Assessment

The primary study outcome was postoperative peak pain level as measured using the NRS immediately before administering rescue fentanyl, which ranged from 0 (indicating no pain) to 10 (indicating severe pain), assessed 1 h postoperatively in the PACU. The pain was evaluated in three specific areas: in the RUQ, representing visceral pain from the gallbladder and surrounding tissues; at the umbilicus, representing parietal pain from the skin incision; and at the shoulder, representing referred pain due to irritation of the phrenic nerve and diaphragm. We also tracked the total amount of rescue fentanyl provided to each group in both the PACU during the first hour postoperatively and the ward during the first 24 h postoperatively.

### 2.5. Clinical Variables

#### 2.5.1. Pre- and Intraoperative Variables

The preoperative variables examined in this study were sex (female), age, body mass index (BMI), ASA physical class, the presence of diabetes mellitus (DM) and hypertension (HBP), hemoglobin levels, white blood cell count, and aspartate and alanine aminotransferase. The intraoperative variables considered included the duration of the operation and the dose of rescue ephedrine administered.

#### 2.5.2. Postoperative Quality of Recovery Assessment

The Korean version of the Quality of Recovery-15 (QoR-15K) questionnaire was used to evaluate the quality of postoperative functional recovery on postoperative day 1. The score for each dimension of the questionnaire was obtained as the sum of the scores of individual items, as follows: physical comfort, items 1–4 and 13; physical independence, items 5 and 8; psychological support, items 6 and 7; emotional state, items 9, 10, 14, and 15; and pain, items 11 and 12 [26].

#### 2.5.3. Postoperative Complications

On the first postoperative day, potential complications associated with nefopam were monitored, including dizziness, sweating, and tachycardia. Additionally, potential complications related to the RSB were monitored, including vascular injury, hematoma, bowel injury, and extrarectus sheath injection.

### 2.6. Sample Size and Statistical Analysis

The study sample size was calculated retrospectively based on 20 single-port RALC cases. The average NRS pain score in the RUQ was 7.3 for patients not receiving nefopam, compared to 4.5 for those who did. To achieve 80% statistical power and maintain a 5% type I error rate with a standard deviation of 3.1, a minimum of 20 patients was required in each group. Factoring in an anticipated 10% dropout rate, this study aimed to enroll 44 patients to ensure the findings’ robustness and reliability.

The Shapiro–Wilk test assessed the normality of the data distribution. For normally distributed data, unpaired *t*-tests were used for comparisons, while the Mann–Whitney *U*-test was used for non-normally distributed data. Categorical data were analyzed using Pearson’s *χ*^2^ test or Fisher’s exact test as appropriate. Results are presented as means ± standard deviations or numbers (%) where suitable. *p*-values of less than 0.05 were considered statistically significant. The statistical analysis was conducted using SPSS for Windows (ver. 24.0; IBM, Armonk, NY, USA).

## 3. Results

The final study cohort consisted of 40 participants, with an average age of 48.3 ± 10.7 years and a BMI of 26.2 ± 4.1 kg/m². Among them, 24 were female (60.0%). Regarding ASA physical status, 14 participants (35.0%) were classified as ASA I and 26 participants (65.0%) as ASA II. The prevalence of DM was 12.5% (*n* = 5), and 27.5% (*n* = 11) had HBP. The preoperative laboratory values included an average hemoglobin level of 13.7 ± 1.5 g/dL, a white blood cell count of 7.2 ± 2.3 × 10^9^/L, an aspartate aminotransferase (AST) level of 26.0 ± 12.4 IU/L, and an alanine aminotransferase (ALT) level of 37.4 ± 50.3 IU/L.

**Figure 2:**
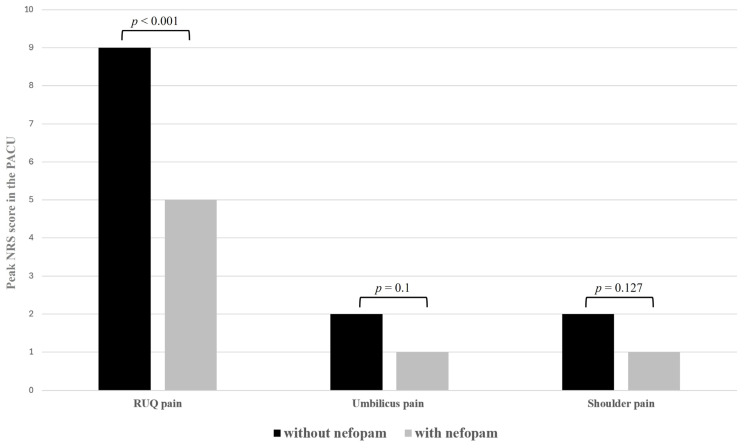
Comparison of RUQ, umbilicus, and shoulder pain between patients receiving nefopam and those not receiving nefopam.

**Figure 3:**
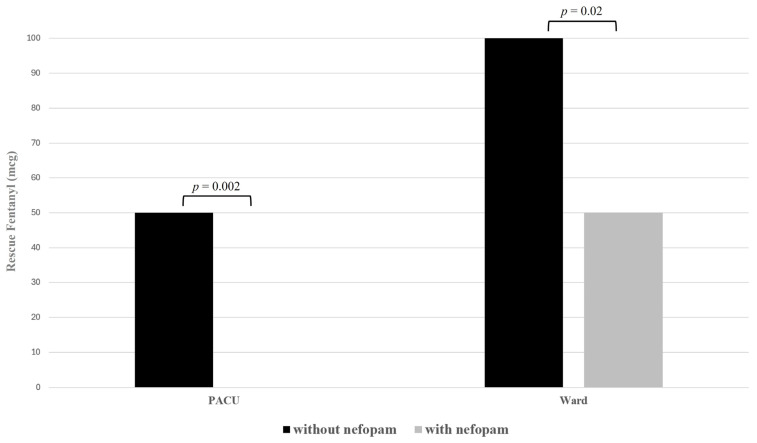
Comparison of rescue fentanyl administration in the PACU and ward between patients receiving nefopam and those not receiving nefopam.

The pre- and intraoperative variables were similar between the patients administered nefopam and those who were not (Table 1). In terms of postoperative pain outcomes, the NRS score for RUQ pain was higher in the patients who did not receive nefopam compared to those who did, while the pain scores for the umbilicus and shoulder in the PACU were comparable between groups (Figure 2 and Figure 3; Table 2). The requirement for rescue fentanyl was more significant in patients not treated with nefopam, both in the PACU and in the ward. None of the patients reported experiencing these side effects during the 24 h following surgery.

Regarding the QoR-15K questionnaire results, the subdimension score for nausea and vomiting was better in patients who received nefopam (Table 3), while the total score and other subdimension scores did not differ between the groups.

## 4. Discussion

Nefopam effectively reduced RUQ pain, which typically arises from visceral sources, and lowered opioid use during early postoperative recovery. However, it did not enhance the effectiveness of RSB at alleviating umbilicus (associated with parietal pain) or shoulder (a form of referred pain) pain. Evaluation using the QoR-15K questionnaire 1 day after surgery revealed no significant differences in the overall recovery quality or specific aspects between the groups. Notably, the patients who received nefopam had significantly improved scores related to nausea and vomiting, highlighting its role in reducing early postoperative opioid requirements. This is particularly significant for patients who have undergone a parietal pain block, where nefopam could notably decrease the need for additional opioid analgesics and lessen PONV discomfort.

Previous studies have demonstrated the analgesic efficacy of nefopam in patients who underwent various types of robot-assisted surgeries. Patients who received nefopam during bilateral axillo-breast approach robotic or endoscopic thyroidectomy reported significantly lower postoperative pain scores and required fewer rescue analgesics compared to those receiving saline [27,28,29]. Similarly, in a study involving robotic nephrectomy, nefopam administration significantly reduced catheter-related bladder discomfort postoperatively [28]. Another study comparing continuous lidocaine infusion and nefopam injection to fentanyl-based patient-controlled analgesia (PCA) found comparable pain relief but with a lower incidence of PONV in the nefopam group [27]. Our findings align with these studies, demonstrating that while RSB effectively alleviates parietal pain, incorporating nefopam reduces visceral pain further. However, the combination of nefopam with RSB did not significantly improve the management of umbilical port pain, suggesting that nefopam and RSB, each targeting different pain types, are effective for managing visceral and parietal pain, respectively. 

In contrast to a recent report by Becerra-Bolaños et al., which documented an average postoperative pain intensity of 2.7 ± 2.8 on the NRS [30], our study found higher postoperative pain levels. Several factors might explain this discrepancy. Firstly, the nature of single-port RALC, which involves visceral, parietal, and referred shoulder pain, could inherently result in higher postoperative pain levels compared to the other surgical procedures analyzed in the cited study. Secondly, our methodology involved assessing pain intensity at specific postoperative intervals, including peak pain before rescue fentanyl administration, which may have captured higher pain scores. Additionally, the absence of preemptive analgesia in our protocol aimed to isolate and evaluate the analgesic efficacy of nefopam, possibly leading to higher initial pain reports. Furthermore, cultural and institutional differences in pain perception and reporting behaviors might have contributed to these variations. Understanding these factors underscores the need for standardized pain assessment and management protocols to ensure consistent and reliable postoperative pain data across different surgical settings. 

Recent findings suggest that combining nefopam with other non-opioid analgesics could result in lower pain intensity. A study demonstrated that the combination of nefopam with ketoprofen and acetaminophen significantly improved postoperative pain management and reduced opioid consumption [31]. Patients receiving this multimodal analgesic regimen exhibited lower morphine requirements and better pain control compared to those given fewer or no non-opioid analgesics. These results support the idea that integrating nefopam with other non-opioid analgesics can enhance analgesic efficacy and reduce opioid reliance. However, our study focused on evaluating the opioid-sparing effect of nefopam in patients who received a parietal pain block, such as the RSB, without the addition of other non-opioid medications. Future research is required to investigate the benefits of multimodal pain strategies that include various non-opioid analgesics and nerve blocks, potentially providing even greater improvements in postoperative pain management.

Analyses of nefopam’s impact on PONV and gastrointestinal discomfort, common adverse effects associated with opioids, revealed nuanced outcomes. A meta-analysis and several prospective studies highlight nefopam’s potential benefits and limitations in this context [32,33,34]. One meta-analysis showed that nefopam significantly reduced incidences of nausea, vomiting, and pruritus, suggesting it is advantageous for minimizing opioid-related adverse effects [33]. Prospective studies demonstrated that nefopam reduced the incidence and severity of PONV, particularly when compared to fentanyl [34]. However, another study found no significant difference in PONV incidence between nefopam and fentanyl groups [32]. While nefopam effectively reduces the need for morphine, it can induce nausea in 10–30% of patients, typically after the initial dose [35,36]. These results suggest that nefopam can effectively reduce PONV by decreasing opioid consumption, though its direct antiemetic effects are limited. Balancing nefopam’s morphine-sparing benefits against its potential side effects is crucial for optimal patient care.

There were several limitations to this study. First, our focus was on assessing pain outcomes and opioid consumption only within the first hour after surgery in the PACU. Additional research should examine the effects of nefopam on visceral and parietal pain over a longer duration. However, we were able to evaluate the quality of patient recovery within the first 24 h post-surgery using the QoR-15K questionnaire in addition to measuring opioid administration. Second, many studies have administered 20 mg nefopam, a dosage likely determined based on morphine-equivalent studies [16,17,18]. By contrast, we opted for a 40 mg dose to assess its effectiveness in pain control, suggesting that further research on the appropriate nefopam doses for different types of surgery is necessary. Third, while we investigated the pain control efficacy of nefopam following RSB, research into its interactions with various blocks is needed. This is particularly relevant now that Enhanced Recovery After Surgery (ERAS) protocols are being widely implemented in surgical care [37], indicating that such studies could be valuable in pain management strategies. Fourth, the intraoperative RSB performance could have increased nefopam’s analgesic effect, and the absence of postoperative analgesics could have increased postoperative pain in patients who did not receive intraoperative nefopam.

## 5. Conclusions

Our study adds to the growing body of evidence on the effectiveness of nefopam at reducing postoperative pain, particularly RUQ pain associated with visceral discomfort, and in decreasing opioid dependence immediately after surgery, particularly in patients undergoing single-port RALC with a parietal pain block. While nefopam does not significantly enhance RSB effectiveness in mitigating umbilicus parietal pain or shoulder referred pain, it notably lowers the incidence and severity of nausea and vomiting. This underscores the value of nefopam in opioid-sparing strategies, highlighting its contribution to multimodal pain management approaches. These outcomes suggest nefopam can improve patient recovery experiences by reducing opioid-related side effects, marking a step forward in postoperative care.

## Figures and Tables

**Table 1:** Comparison of the pre- and intraoperative findings between patients with and without nefopam.

Group	Without Nefopam	With Nefopam
*n*	20	20
Preoperative variables		
Sex (female)	11 (55.0%)	13 (65.0%)
Age (years)	47.8 ± 9.2	48.9 ± 12.3
Body mass index (kg/m^2^)	27.2 ± 4.1	25.2 ± 4.0
ASA physical status classification		
I	6 (30.0%)	8 (40.0%)
II	14 (70.0%)	12 (60.0%)
Diabetes mellitus	3 (15.0%)	2 (10.0%)
Hypertension	8 (40.0%)	3 (15.0%)
Laboratory variables		
Hemoglobin (g/dL)	13.9 ± 1.4	13.4 ± 1.5
White blood cell count (×10^9^/L)	7.3 ± 1.9	7.0 ± 2.7
Aspartate aminotransferase (IU/L)	21.5 (16.3–24.8)	26.0 (19.5–33.0)
Alanine aminotransferase (IU/L)	24.5 (13.5–38.8)	24.5 (14.8–35.0)
Intraoperative variables		
Operation time (min)	65.6 ± 13.6	69.6 ± 17.4
Rescue ephedrine dose (mg)	0.0 (0.0–0.0)	0.0 (0.0–0.0)

Abbreviations: ASA, American Society of Anesthesiologists. Values are expressed as means (standard deviation), medians (interquartile), and numbers (percentages).

**Table 2:** Comparison of the postoperative pain outcomes between patients with and without nefopam.

Group	Without Nefopam	With Nefopam	*p*
*n*	20	20	
Peak NRS score in the PACU			
RUQ pain	9 (8–10)	5 (3–6)	<0.001
Umbilicus pain	2 (1–5)	1 (1–2)	0.1
Shoulder pain	2 (1–3)	1 (1–2)	0.127
Rescue fentanyl in the PACU (mcg)	50 (50–100)	0 (0–50)	0.002
Rescue fentanyl in the ward (mcg)	100 (50–100)	50 (25–94)	0.02

Abbreviations: NRS, numeric rating scale; PACU, post-anesthesia care unit; RUQ, right upper quadrant. Values are expressed as medians (interquartile).

**Table 3:** Comparison of the global and subdimension scores from the Quality of Recovery-15 questionnaire on postoperative day 1 between patients with and without nefopam.

Group	Without Nefopam	With Nefopam	*p*
*n*	20	20	
Total QoR-15 score (points)	93 (72–119)	106 (69–134)	0.482
1 Able to breathe easy	9 (5–10)	7 (5–10)	0.813
2 Been able to enjoy food	5 (0–9)	5 (1–10)	0.564
3 Feeling rested	6 (3–9)	8 (5–10)	0.434
4 Have had a good sleep	6 (2–9)	7 (2–10)	0.87
5 Able to look after personal toilet and hygiene unaided	10 (4–10)	10 (4–10)	0.977
6 Able to communicate with family or friends	10 (5–10)	10 (4–10)	0.678
7 Getting support from hospital doctors and nurses	10 (9–10)	10 (9–10)	0.633
8 Able to return to work or usual home activities	5 (2–10)	6 (2–10)	0.858
9 Feeling comfortable and in control	7 (4–9)	8 (5–10)	0.373
10 Having a feeling of general well-being	6 (3–9)	7 (4–10)	0.228
11 Moderate pain	4 (2–6)	4 (2–6)	0.681
12 Severe pain	4 (2–5)	4 (2–10)	0.337
13 Nausea or vomiting	10 (7–10)	10 (10–10)	0.006
14 Feeling worried or anxious	7 (3–10)	9 (5–10)	0.23
15 Feeling sad or depressed	7 (5–10)	10 (4–10)	0.36

Abbreviations: QoR, quality of recovery. Values are expressed as medians (interquartile).

## Data Availability

Data are contained within the article.

## Abbreviations

RALC, single-port robot-assisted laparoscopic cholecystectomy; RSB, rectus sheath block; NRS, numeric rating scale; RUQ, right upper quadrant; QoR-15K, Quality of Recovery-15 questionnaire
